# A Review of FDA-Approved Anti-HIV-1 Drugs, Anti-Gag Compounds, and Potential Strategies for HIV-1 Eradication

**DOI:** 10.3390/ijms25073659

**Published:** 2024-03-25

**Authors:** Belgin Sever, Masami Otsuka, Mikako Fujita, Halilibrahim Ciftci

**Affiliations:** 1 Department of Pharmaceutical Chemistry, Faculty of Pharmacy, Anadolu University, Eskisehir 26470, Türkiye; belginsever@anadolu.edu.tr; 2 Medicinal and Biological Chemistry Science Farm Joint Research Laboratory, Faculty of Life Sciences, Kumamoto University, Kumamoto 862-0973, Japan; motsuka@gpo.kumamoto-u.ac.jp; 3 Department of Drug Discovery, Science Farm Ltd., Kumamoto 862-0976, Japan; 4 Department of Bioengineering Sciences, Izmir Katip Celebi University, Izmir 35620, Türkiye

**Keywords:** HIV-1, AIDS, FDA, combination antiretroviral therapy, latent reservoirs, apoptosis, drug resistance, anti-HIV-1 drug design and discovery

## Abstract

Acquired immunodeficiency syndrome (AIDS) is an enormous global health threat stemming from human immunodeficiency virus (HIV-1) infection. Up to now, the tremendous advances in combination antiretroviral therapy (cART) have shifted HIV-1 infection from a fatal illness into a manageable chronic disorder. However, the presence of latent reservoirs, the multifaceted nature of HIV-1, drug resistance, severe off-target effects, poor adherence, and high cost restrict the efficacy of current cART targeting the distinct stages of the virus life cycle. Therefore, there is an unmet need for the discovery of new therapeutics that not only bypass the limitations of the current therapy but also protect the body’s health at the same time. The main goal for complete HIV-1 eradication is purging latently infected cells from patients’ bodies. A potential strategy called “lock-in and apoptosis” targets the budding phase of the life cycle of the virus and leads to susceptibility to apoptosis of HIV-1 infected cells for the elimination of HIV-1 reservoirs and, ultimately, for complete eradication. The current work intends to present the main advantages and disadvantages of United States Food and Drug Administration (FDA)-approved anti-HIV-1 drugs as well as plausible strategies for the design and development of more anti-HIV-1 compounds with better potency, favorable pharmacokinetic profiles, and improved safety issues.

## 1. Introduction

Human immunodeficiency virus (HIV), an enveloped virus with a diploid, positive-sense, and single-stranded RNA, belongs to the *Lentivirus* genus within the *Orthoretrovirinae* subfamily of the *Retroviridae* family. HIV-1 and HIV-2 are the two main types of HIV and HIV-1 is responsible for the majority of infections across the globe [1,2,3,4]. Acquired immunodeficiency syndrome (AIDS), stemming mainly from HIV-1, is one of the most serious hurdles throughout the world. The last updated World Health Organization (WHO) report indicated that globally about 39.0 million people acquired HIV-1 and 630,000 people died from HIV-1-dependent problems at the end of 2022 [5]. Furthermore, HIV-1/AIDS provokes opportunistic infections including tuberculosis, cryptococcal infection, histoplasmosis, and severe bacterial infections; hepatitis B, and C co-infections [6,7,8]; and comorbidities such as cardiovascular, kidney, and liver disorders and cancer [9]. In the near future, COVID-19 and monkeypox virus infections could be possible challenges for clinicians and people with HIV-1 [10,11].

The typical flu symptoms may appear at 2–6 weeks after acute HIV-1 infection, then it can remain silent for years without any symptoms and testing is the only solution to diagnose HIV-1. The “eclipse period” or “window period” is known as the time between exposure and confirmed identification of infection. It is hard to predict this period due to obscurity of the exact time of the exposure, although recent HIV-1 testing technologies applied in clinic are able to identify some parameters, including viral DNA, capsid (CA) protein, HIV-1 antibodies, and antibody/antigen in combination [12,13,14]. 

**Figure 1:**
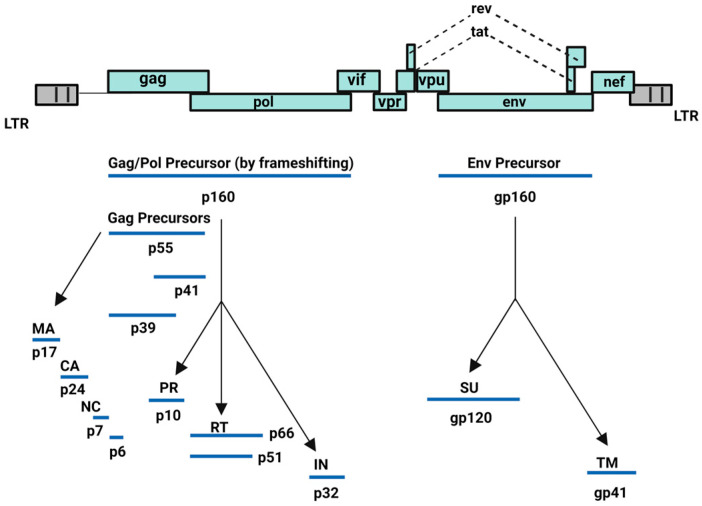
HIV-1 encoded proteins [15]. Image created with BioRender.com.

HIV-1 is capable of converting its RNA into DNA through reverse transcriptase or RNA-dependent DNA polymerase enzyme and subsequently, integrating its viral DNA into the host cell DNA through integrase enzyme. HIV-1 incorporates nine genes: structural (*gag*, *pol*, and *env*), regulatory (*tat*, *rev*), and accessory (*vif*, *vpr*, *vpu*, *nef*) genes. The group-specific antigen (*gag*) gene encodes for Gag polyprotein precursor (Pr55^Gag^: Gag), whereas the Gag-Pol polyprotein precursor is expressed via ribosomal frameshifting between the *gag* and *pol* genes. Finally, the *env* gene encodes for the viral envelope glycoproteins (Figure 1) [15]. All these proteins orchestrate the viral cell cycle of HIV-1 leading to redundancy in infected host cells, mainly CD4^+^ T cell lymphocytes [15,16,17,18,19,20,21].

**Figure 2:**
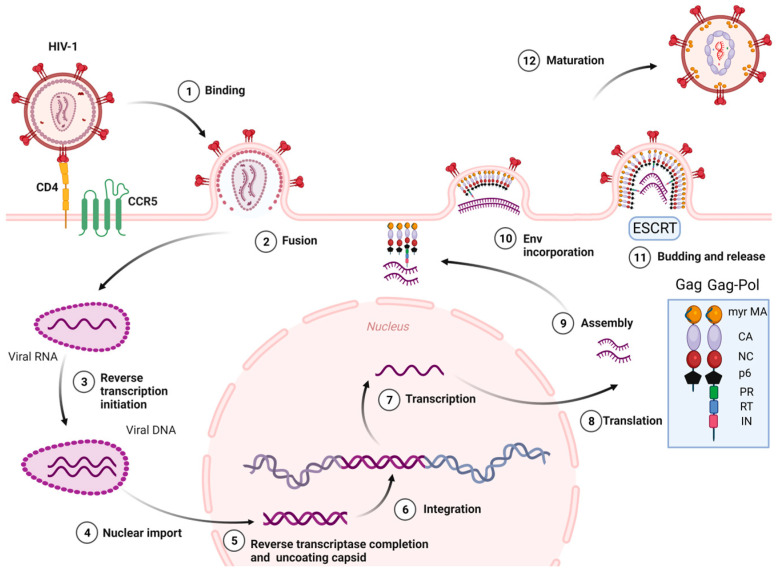
HIV-1 life cycle. Myr-MA: Myristoylated-Matrix, CA: Capsid, NC: Nucleocapsid, PR: Protease, RT: Reverse Transcriptase, IN: Integrase, ESCRT: Endosomal sorting complexes required for transport. Image created with BioRender.com.

The stages of the HIV-1 cell cycle are: (i) Attachment, fusion and entry: The attachment and fusion between the HIV-1 envelope and the cell surface of CD4^+^ cells occur through viral glycoproteins 120 and 41 (gp120 and gp41), the subunits of Env glycoprotein, and the host chemokine receptors (CCR5 or CXCR4) located on the surface of CD4^+^ cell, respectively. Then, HIV-1 enters into the CD4^+^ cell. (ii) Reverse transcription of viral RNA: The viral RNA is transcribed to viral DNA by reverse transcriptase enzyme, located within the CA, which occludes exposure of the viral genome to host proteins. This process is known to start in the cytoplasm. Recent studies [22] have reported that cDNA synthesis ends inside the nucleus. (iii) Integration: The HIV-1 DNA is integrated into the cellular DNA by integrase enzyme for its further replication, transcription, and translation into viral proteins. (iv) Assembly, budding, release, and maturation: (1) Gag proteins move to the plasma membrane, form hexameric subunits, and assemble into immature lattice; (2) RNA dimers are recruited to the assembly region and Env trimers are included in the budding particles; (3) membrane sequestration and release of virus particles from the cell surface is driven by the endosomal sorting complexes required for transport (ESCRT) and the Gag-p6 domain; (4) subsequent viral protease-mediated cleavage of Gag and Gag-Pol polyprotein occurs to constitute mature structural and viral proteins, self-assembly and generation of mature CA protein, and the final formation of mature particles, which are able to infect other immune cells (Figure 2) [23,24,25,26,27,28]. 

After the discovery of the first anti-HIV-1 drug zidovudine in 1987, numerous agents acting as inhibitors of reverse transcriptase, integrase, entry (attachment, fusion, post-attachment), protease, and CA or pharmacokinetic enhancers have rendered the impact of HIV-1/AIDS from a devastating fatal disorder to a manageable chronic infection [29,30,31,32]. The combined use of these agents, entitled combination antiretroviral therapy (cART, ART, formerly highly active antiretroviral therapy (HAART), cocktail therapy), marked a watershed moment to ameliorate the prognosis of the illness [33,34,35]. The three-drug regimen with a dual nucleoside analogue reverse transcriptase inhibitor (NRTIs) backbone and a core drug from integrase strand transfer inhibitors (INSTIs), boosted protease inhibitors, attachment inhibitor, fusion inhibitor, or non-nucleoside reverse transcriptase inhibitor (NNRTI) has become the mainstay for the initial HIV-1 treatment [36,37,38,39]. Moreover, the once-daily fixed-dose three-drug combination in a single tablet regimen is one of the milestones for HIV-1/AIDS treatment, facilitating a reduced pill burden and dosing frequency and improved adherence [40,41].

The major obstacles of the current therapy are persistent latent reservoirs, adherence challenges, drug resistance, limited treatment options for multi-class resistance, drug–drug interactions, limited availability of drug in poor countries, and a high cost of the lifetime treatment regimen. There are also concerns about adverse effects such as cardiovascular events, insulin resistance and type II diabetes, renal dysfunction, hepatotoxicity, lipodystrophy, gastrointestinal toxicities (nausea, vomiting, and diarrhea), rash, chronic pain, and central nervous system (CNS) toxicities [42,43,44,45,46]. The other factors influencing HIV-1 treatment in particular include continued viral transmission among people who cannot reach testing or treatment due to barriers such as stigma, discrimination, lack of confidentiality, or gender-based disadvantages [13,47,48]. Mother-to-child transmission during pregnancy and breastfeeding is also another point that caution is required for the implementation of cART to enhance safety of mothers with HIV-1 infection and their exposed fetuses and children [49].

The current treatment is lifelong, which paves the way for HIV-1 drug resistance and poor medication compliance. Therefore, to develop new inhibitors with novel mechanisms of action is crucial. This review focuses on the evaluation of pearls and pitfalls and updates in the use of approved anti-HIV-1 drugs and bona fide approaches for HIV-1 eradication. 

## 2. United States Food and Drug Administration (FDA)-Approved Drugs for HIV-1 Treatment

### 2.1. Reverse Transcriptase Inhibitors

Reverse transcriptase enzyme has two distinct activities: as a DNA polymerase, which catalyzes the synthesis of a double-stranded proviral DNA from a single-stranded viral RNA, and as an endonuclease, which exerts RNase H activity by hydrolyzing the RNA strand in an RNA/DNA hybrid. The HIV-1 reverse transcriptase enzyme incorporates 66 kDa (p66) and 51 kDa (p51) subunits. The p66 subunit takes after the closed right hand with palm, fingers, thumb, and connection subdomains and contains the polymerase and the RNase H active sites [50,51,52,53].

**Figure 3:**
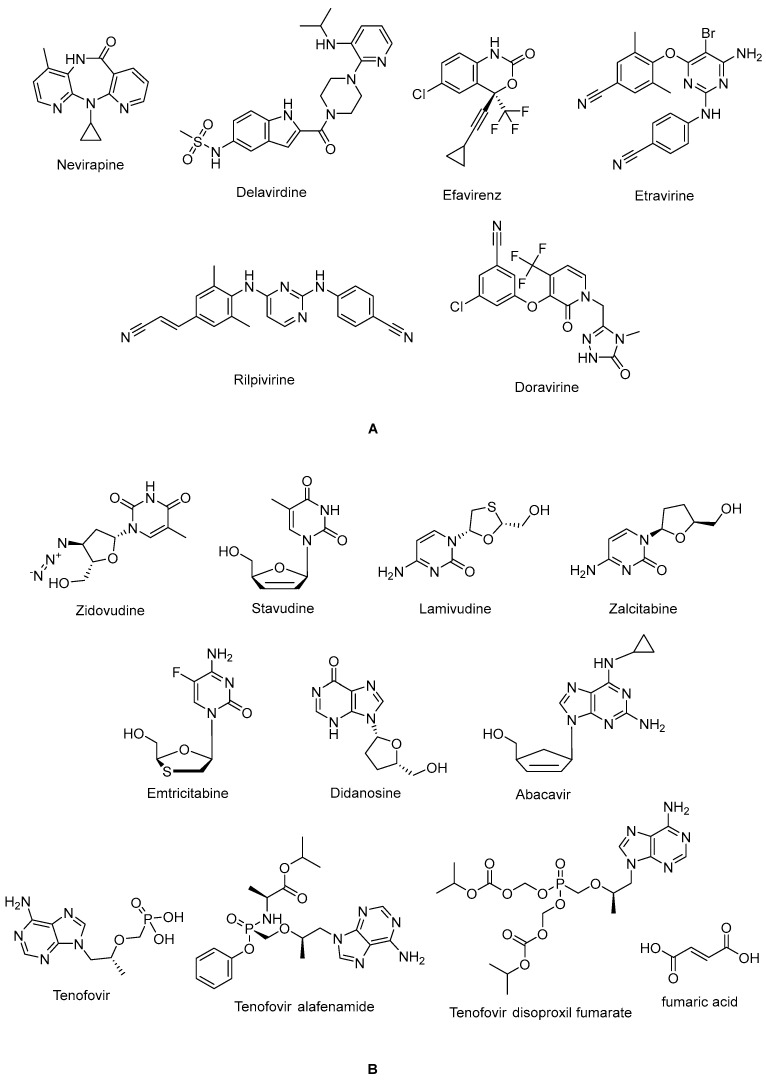
The chemical structures of FDA-approved NNRTIs (**A**) and NRTIs (**B**).

The non-nucleoside reverse transcriptase inhibitor (NNRTI) site is linked with the palm subdomain to a great extent and is far from the polymerase active site; thus, it serves as a non-competitive inhibitor, in contrast to nucleoside reverse transcriptase inhibitors (NRTIs), which compete at the active site. Therefore, non-nucleoside reverse transcriptase inhibitors (NNRTIs) constitute compounds possessing diverse chemical structures, whereas NRTIs are pyrimidine/purine (nucleoside) or nucleotide analogues. The first-generation NNRTIs nevirapine (Viramune^®^) (Figure 3) and delavirdine (Rescriptor^®^) (Figure 3) are prone to be affected by single point mutations. Delavirdine was discontinued due to severe adverse effects and nevirapine, a derivative of dipyridodiazepinone, is not recommended anymore due to side effects such as hepatotoxicity and rash, though it is a potent and easy-to-take antiretroviral drug [54]. On the other hand, efavirenz (Sustiva^®^) (Figure 3) is stronger against drug resistance mutations, but side effects such as neuropsychiatric disorders and poor liver function were manifested. Efavirenz was one of the first options appropriate for once-daily use [55,56]. Etravirine (Intelence^®^) (Figure 3), rilpivirine (Edurant^®^) (Figure 3), diarylpyrimidine derivatives, and doravirine (Pifeltro^®^) (Figure 3) are second-generation inhibitors. Etravirine is an alternative option for HIV-1-infected people with first-generation NNRTI resistance, but its bitter taste and twice-daily dosing impede its clinical use [57]. Rilpivirine was demonstrated to be potent with less off-target effects in HIV-1-infected patients compared to efavirenz [58].

Rilpivirine-containing regimens are expedient in various terms, such as a single-tablet formulation with tenofovir and emtricitabine (NRTIs). Rilpivirine is a component of co-formulations (Complera^®^, Odefsey^®^) with emtricitabine (Figure 3) along with tenofovir diproxil fumarate (Figure 3) or tenovofir alafenamide (Figure 3) [59,60]. Since the bioavailability of rilpivirine is boosted under acidic conditions, it must not be administered with proton pump inhibitors concomitantly. Cytochrome p450 family 3 subfamily A (CYP3A) enzyme inducers such as anticonvulsants (carbamazepine, phenobarbital, and phenytoin) and antimycobacterial agents (rifampicin) may cause lower anti-HIV-1 activity [58]. Doravirine, a pyridone derivative, exhibited a broad spectrum of antiviral activity towards clinically relevant mutant viruses. It was also found to be beneficial with improved pharmacokinetic parameters for alternative use in patients, with the advantages of less propensity for resistance and toxicity. Doravirine is on the market as a fixed-dose combination tablet with lamivudine and tenofovir disoproxil fumarate (Delstrigo^®^). This combination requires attention for patients with HIV-1–hepatitis B co-infection [61,62,63,64]. 

HIV-1 NRTIs mimic and compete with natural deoxynucleotide triphosphates (such as dTTP, dCTP, dGTP, and dATP) for incorporation at the polymerase active site. Most approved NRTIs with a missing hydroxy group at the 3′ position are incorporated into proviral DNA as chain terminators by reverse transcriptase since they are substrates for reverse transcriptase, which converts them to the corresponding 5′-triphosphates. Nucleoside analogues of NRTIs are phosphorylated to achieve their active di- or tri-phosphate anabolites by host enzymes within a cell. However, tenofovir disoproxil fumarate (Figure 2), a nucleotide analogue, is found in a monophosphate form and two additional phosphorylation steps are required to run into its active form. Since the common structure of NRTIs is a trigger for the development of resistance to NRTIs, they are used in combination with other NRTIs and NNRTIs rather than as a single agent nowadays [30,65,66,67,68]. 

NRTIs are the key backbones of combination regimens according to the recent guidelines. Zidovudine (Retrovir^®^) (Figure 3), the first approved anti-HIV-1 drug, and stavudine (Zerit^®^) (Figure 3) are thymidine analogues. The exploration of zidovudine as an anti-HIV agent is also the first example of drug repositioning in the history of medicinal chemistry, as it was initially developed as an anticancer agent [69]. The use of stavudine is not recommended by the WHO, as it increases the lipoatrophy risk, along with other side effects. Lamivudine (Epivir^®^) (Figure 3), zalcitabine (Hivid^®^) (Figure 3), and emtricitabine (Emtriva^®^) (Figure 3) are cytosine analogues. Renal dysfunction is a challenging point for the use of lamivudine and the dosage must be adjusted for patients with renal impairment. Lamivudine was licensed for the treatment of HIV-1 and hepatitis B virus infections in 1995 and 1998, respectively. Lamivudine, a key component in HIV-1 treatment, is highly recommended in almost all first-line and a majority of second-line combination regimens thanks to its remarkable efficacy and safety profile. The combinations of tenofovir disoproxil fumarate with lamivudine (Cimduo^®^) or emtricitabine (Truvada^®^) are also recommended as a first-line treatment for patients co-infected with HIV-1 and hepatitis B virus [70]. 

Emtricitabine received FDA approval in 2003 for use in anti-HIV-1 treatment. Emtricitabine, the most commonly prescribed anti-HIV-1 drug, is a fluorinated derivative of lamivudine, and they share the same sugar configuration [71]. Zalcitabine is not recommended for use in many countries due to high mitochondrial toxicity. Didanosine (Videx^®^) (Figure 3) and abacavir (Ziagen^®^) (Figure 3) are adenosine and guanosine analogues, respectively. Didanosine is not prescribed, as it has toxicity, efficacy, formulation, and drug interaction issues. Abacavir has a higher risk of cardiovascular diseases. Therefore, abacavir is also available in coformulated tablets that contain other antiretrovirals such as lamivudine (Kivexa^®^, Epzicom^®^), lamivudine plus dolutegravir (INSTI) (Triumeq^®^), and lamivudine plus zidovudine (Trizivir^®^) [72]. Given that tenofovir displays poor cellular absorption and oral bioavailability owing to the negative charges on the phosphonate moiety, the fumarate salts of tenofovir disoproxil (Viread^®^) and tenofovir alafenamide (Figure 3) are prepared as tenofovir prodrugs. Tenofovir alafenamide (Vemlidy^®^), which was developed to deal with the renal and bone toxicity of tenofovir disoproxil fumarate, has superior potential and safety properties over other NRTIs. Moreover, HIV-1 treatment guidelines mainly recommend its combination with emtricitabine. On the other hand, tenofovir disoproxil fumarate/emtricitabine is the recommended oral pre-exposure prophylaxis regimen for all populations at risk [65,73,74,75,76].

### 2.2. Protease Inhibitors

Protease enzymes drive the catalysis of the hydrolysis of polypeptide bonds. The HIV-1 protease is a homodimeric aspartyl protease, which can cleave HIV-1 precursor protein or polyprotein involving structural proteins (Gag proteins: matrix (MA), CA, nucleocapsid (NC), p6, and enzyme products) and viral enzymes (Pol proteins: protease, reverse transcriptase, RNase H, and integrase). The Gag and Gag-Pol viral polyprotein are associated with efficient viral assembly, genome packaging, formation of immature viral particles, budding, release from the cell, and maturation in order to infect new host cells. The timing of the release of HIV-1 protease from the Gag-Pol polyprotein is crucial for accomplished maturation process and, consequently, HIV-1 infectivity [77,78,79,80,81]. 

**Figure 4:**
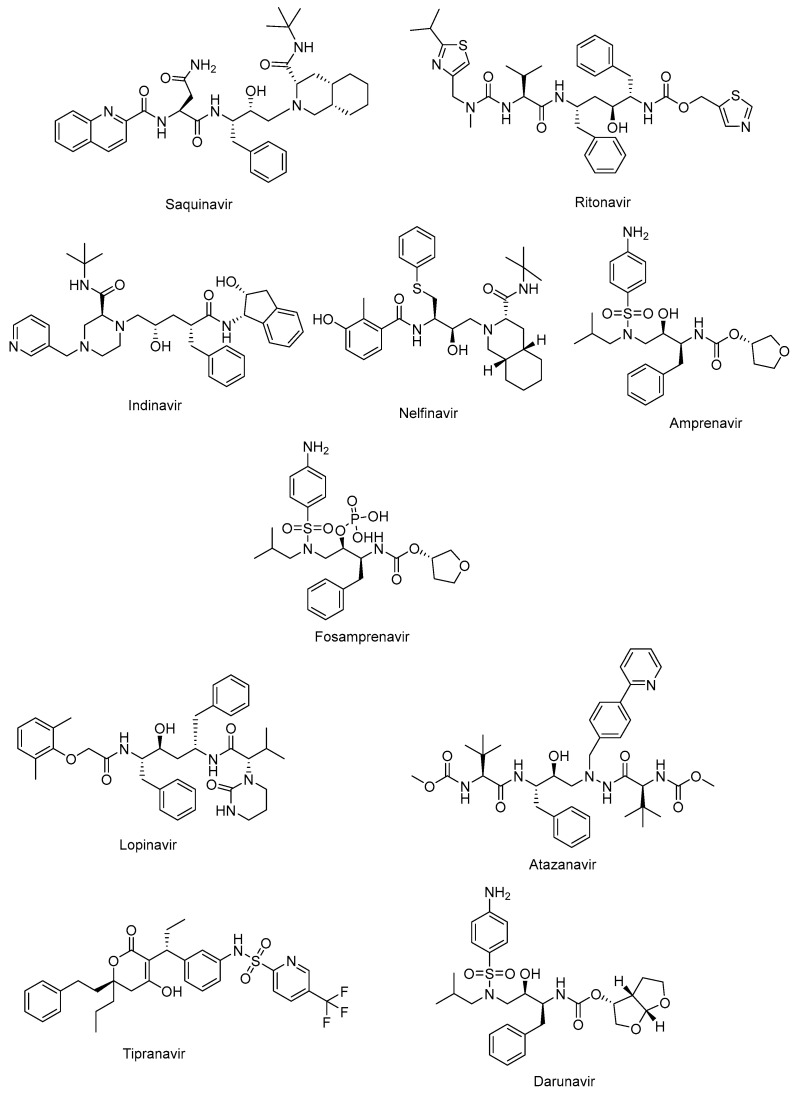
The chemical structures of FDA-approved first- and second-generation protease inhibitors.

The active site of protease is a catalytic Asp-Ser-Gly triad that enables the nucleophilic attack of water onto the scissile amide bond of natural substrate for successful cleavage. The protease inhibitors, non-cleavable transition state isosteres, can compete with the natural substrate by a functional group acting as a scissile amide bond mimetic [82,83]. The protease inhibitors were initially developed based on a “structure-informed” strategy aiming at HIV-1 protease inhibition and successive inhibition of HIV-1 replication [84]. The first protease inhibitors, including saquinavir (Invirase^®^) (Figure 4), ritonavir (Norvir^®^) (Figure 4), indinavir (Crixivan^®^) (Figure 4), nelfinavir (Viracept^®^) (Figure 4), amprenavir (Agenerase^®^) (Figure 4), and fosamprenavir (Lexiva^®^) (Figure 4), shared some similar structural properties and binding patterns, resulting in possible cross-resistance and common severe side effects. Some pharmacokinetic challenges were also encountered with the first HIV-1 protease inhibitors, such as poor oral bioavailability, extensive binding to plasma proteins, and rapid elimination [85]. The second generation of protease inhibitors, including lopinavir (Kaletra^®^), atazanavir (Reyataz^®^), tipranavir (Aptivus^®^), and darunavir (Prezista^®^), are currently recommended for use as second-line regimens for patients with HIV-1 who have received no benefit from the first-line therapy with integrase and reverse transcriptase inhibitors. They are available in a fixed-dose regimen in combination with emtricitabine and tenofovir [86]. 

Lopinavir (Figure 4) in combination with ritonavir was approved by the FDA in 2004 for the treatment of HIV infections. Ritonavir promotes the increased bioavailability of lopinavir as a pharmacoenhancer. Atazanavir (Figure 4), a hydroxyethyl hydrazine aza-peptide inhibitor, is a well-tolerated effective treatment for HIV-1 patients with an extended half-life and fewer side effects and resistance features [83]. Atazanavir is also reported to alleviate competitive bilirubin binding and the relative possible risk of cardiovascular disorders [87]. Tipranavir (Figure 4) is a sulfonamide-based dihydropyrone revealing a different binding profile than the other HIV-1 protease inhibitors, making it stronger for the development of resistance. Tipranavir was found to be effective for both naïve and highly experienced antiretroviral treatment patients, but its therapeutic effect is limited due to some harsh unwanted effects, including intracranial hemorrhage and hepatotoxicity [88,89]. Darunavir (Figure 4) is a small molecule that is capable of impeding the dimerization of HIV protease and its catalytic activity. Darunavir is effective for the treatment of both naïve and experienced HIV-1-infected patients. Darunavir has a high genetic barrier to resistance development and favorable safety profile [90,91]. 

### 2.3. Entry Inhibitors

For the entry process, all enveloped viruses perform a multistep fusion machinery in which their lipid bilayers and the host cell membrane reunite. The HIV-1 Env glycoprotein (gp160) is cleaved to form a gp120-gp41 heterodimer. The three surface gp120 subunits that are non-covalently bound to three transmembrane gp41 subunits constitute a functional trimeric envelope spike on the virion surface, which is essential for host cell recognition and membrane fusion. In the attachment process, gp120 attaches to the primary receptor CD4 antigen on the host cell. This attachment results in structural changes in gp120 and it interacts with a co-receptor (CCR5 or CXCR4). Co-receptor binding triggers the fusion mediated by gp41. During the fusion, the gp41 forms a stable six-helix bundle involving N-terminal heptad repeat (NHR) coiled coils and three C-terminal heptad repeat (CHR) helices packing into the hydrophobic NHR grooves as antiparallel [92,93,94,95,96,97]. 

The HIV-1 entry inhibitors were developed based on three categories: gp120-CD4 binding inhibitors, gp120-co-receptor binding inhibitors, and fusion inhibitors. Enfuvirtide (Fuzeon^®^), the first approved peptide HIV-1 inhibitor, acts as an HIV-1 fusion inhibitor binding to a region of the gp41 of HIV-1. Enfuvirtide was found to be effective in clinical trials against resistant HIV-1 strains. The disadvantages of the treatment with enfuvirtide are its short half-life, which needs long-term application, uncomfortable subcutaneous administration that causes injection-site reactions, and high prices [98,99].

**Figure 5:**
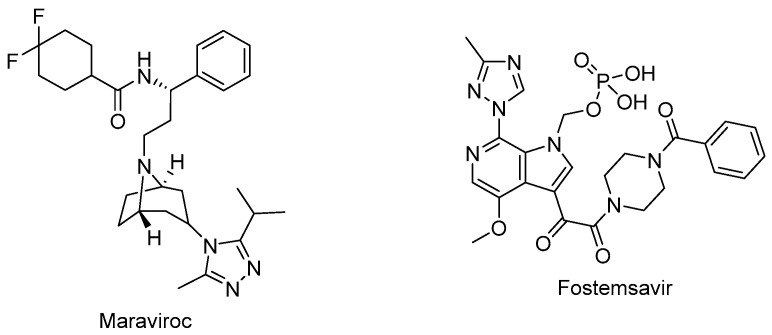
The chemical structures of maraviroc (CCR5 antagonist) and fostemsavir (attachment inhibitor).

Maraviroc (Selzentry^®^) (Figure 5) is the first-in-class and only FDA-approved drug as an inhibitor of CCR5 for the treatment of R5-tropic HIV-1 infected patients. It was discovered as the end product of a high-throughput screening and medicinal chemistry program. Maraviroc was found to be effective at disrupting gp120-CCR5 binding and subsequently, membrane fusion events necessary for HIV-1 entry into cells. Maraviroc showed potent anti-HIV-1 activity, a low level of resistance, favorable pharmacological properties, and mild-to-moderate hepatic and renal disorders [100,101,102,103].

Ibalizumab (Trogarzo^®^) is the first humanized IgG4 monoclonal antibody and CD4-mediated post-attachment inhibitor for the treatment of HIV-1 infection. Ibalizumab shows its effects by binding to the D2 immunoglobulin domain of cell-surface glycoprotein CD4. Ibalizumab, in combination with other antiretroviral(s), was approved by the FDA in 2018 for the treatment of adults with multidrug-resistant HIV-1 infection. Ibalizumab is a valuable option with a high potency and a favorable pharmacokinetic profile, notwithstanding the necessity of additional studies and long-term post-marketing data [104,105,106,107,108].

Fostemsavir (Rukobia^®^) (Figure 5) was approved by the FDA in 2020 for the treatment of multidrug-resistant HIV-1 infection of heavily treatment-experienced adults whose previous HIV-1 therapy was unsuccessful owing to resistance, intolerance, or safety considerations. Fostemsavir, a phosphonooxymethyl prodrug of temsavir, is converted to its active metabolite by alkaline phosphatase. Temsavir prevents attachment and subsequent entry into the host cell via binding to gp120, holding gp120 in the conformational state, and hampering the initial interaction with surface receptors on CD4^+^ cells. Fostemsavir showed good efficacy and a safety profile in patients with multidrug-resistant HIV-1 infection. Moreover, it revealed no in vitro cross-resistance with other classes of entry inhibitors such as ibalizumab, maraviroc, or enfuvirtide. Fostemsavir can also be used for HIV-1 CCR5/CXCR4 tropism. Possible drug–drug interactions can occur during coadministration with strong CYP3A inducers [109,110,111,112,113].

### 2.4. HIV-1 Integrase Inhibitors

The HIV-1 intasome is a massive nucleoprotein complex containing the integrase protein and the ends of the viral DNA obtained by reverse transcription. The integration process is crucial for viral replication and HIV-1 integrase strand-transfer inhibitors (INSTIs) target the intasomes to halt the integration and subsequent replication processes by binding to the catalytic site of integrase. This catalytic site of integrase is engaged with covalent bonds with the phosphodiester backbone of DNA through divalent cations, metals such as Mg^2+^, required for integrase catalytic reactions. The INSTIs demonstrate a familiar mode of action, whereas they generally possess distinct pharmacokinetic profiles, resulting in different dose frequency, combinations, and drug–drug interactions. INSTIs are current recommended components of frontline and drug-switch cART formulations [114,115,116,117].

**Figure 6:**
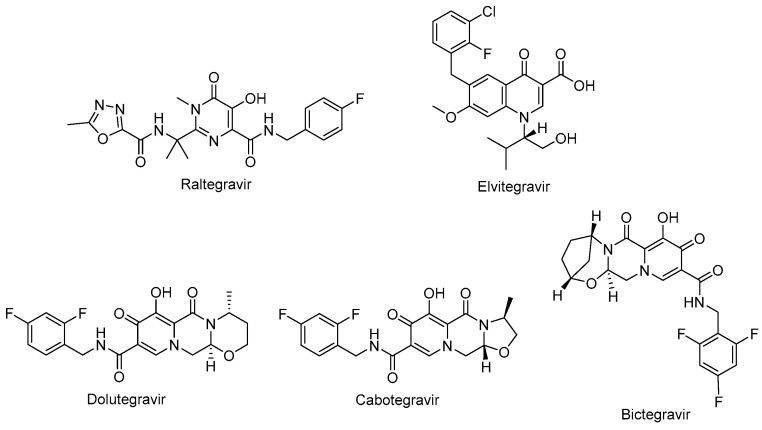
The chemical structures of FDA-approved first- and second-generation INSTIs.

Raltegravir (Isentress^®^) (Figure 6) and elvitegravir (Vitekta^®^) (Figure 6) are first-generation INSTIs. Raltegravir is the first approved INSTI blocking the formation of the covalent bonds. Patients with HIV-1 have experienced a well-tolerated, safe, and potent antiretroviral treatment with raltegravir-based regimens. One of the advantages of raltegravir is glucuronidation metabolism instead of hepatic metabolism, leading to fewer observed drug–drug interactions [118,119]. Elvitegravir was then designed and obtained bearing a coplanar monoketo acid motif in 4-quinolone-3-carbocyclic acid as a bioisostere of a diketo acid motif, of which raltegravir also contains a structural similar moiety, with a pyrimidinone carboxamide [120]. A fixed-dose combination of elvitegravir with cobicistat, emtricitabine, and tenofovir disoproxil fumarate (Stribild^®^) and tenofovir alafenamide instead of tenofovir disoproxil fumarate (Genyova^®^) was approved for the treatment of HIV-1 infection. Dolutegravir (Tivicay^®^) (Figure 6), cabotegravir (Vocabria^®^) (Figure 6), and bictegravir (Figure 6) are second-generation INSTIs. Based on the reports of current guidelines, the combinations of bictegravir, dolutegravir, or raltegravir plus two NRTIs are recommended as initial therapies [74]. Dolutegravir and cabotegravir are bicyclic carbamoyl pyridone analogs using a two-metal chelation model of the integrase catalytic active site [121]. Dolutegravir exhibited a significant activity against HIV-1 isolates with a higher barrier to resistance development compared to raltegravir and elvitegravir. Dolutegravir has several advantages, including favorable pharmacokinetic properties such as a long half-life and once-daily dosing without the need of a pharmacokinetic enhancer or commitment to meal time. The fixed-dose combination of dolutegravir with lamivudine in a single-tablet regimen (Dovato^®^) is confirmed to be effective and well-tolerated for adolescents and adults with HIV-1 infection [122,123,124]. Dolutegravir is also highly recommended by the WHO in the initial therapy combined with NRTI or NNRTI and for use in pregnant women and women with child-bearing potential in second-line treatment. Dolutegravir plus either tenofovir disoproxil fumarate/emtricitabine or tenofovir alafenamide/emtricitabine is recommended as a safe option during pregnancy [74,114,125]. 

The oral tablet form of dolutegravir is available on the market and other long-acting injectables, implants, nasal, and vaginal formulations were found to be beneficial for HIV-1 treatment in preclinical studies [126]. Cabotegravir received approval from the FDA for HIV-1 pre-exposure prophylaxis as the first long-acting injectable medication. It was announced that it is safe and effective for people carrying important HIV-1 infection risk [127,128,129,130]. Bictegravir, structurally derived from dolutegravir, especially inhibited HIV-1 integrase with an IC_50_ value of 7.5 nM compared to dolutegravir and elvitegravir with IC_50_ values of 7.4 nM and 8.4 nM, respectively [131,132]. The advantage of bictegravir over cabotegravir and dolutegravir is its integration into fixed-dose combinations for higher potency and less drug resistance [133]. 

### 2.5. CA Inhibitor

**Figure 7:**
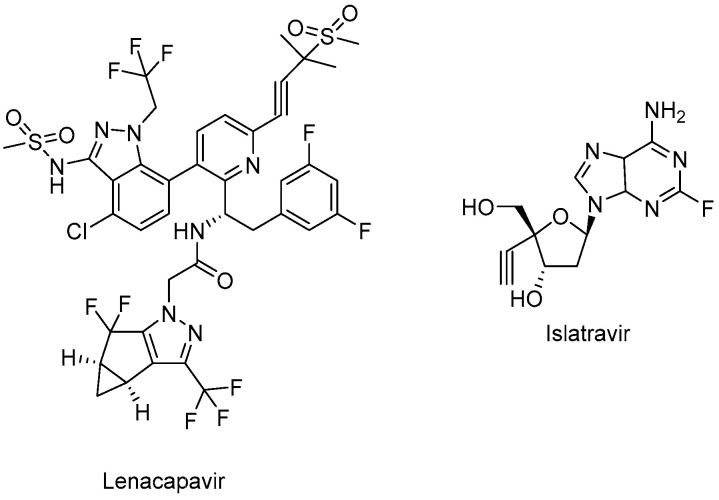
The chemical structures of lenacapavir (CA inhibitor) and islatravir (NRTTI).

Lenacapavir (GSK-6207) (Sunlenca^®^) (Figure 7), developed by Gilead Sciences Inc., was approved by the FDA (2022) for clinical use in combination with other antiretroviral(s) in heavily treatment-experienced patients with multidrug-resistant HIV-1 infection. Lenacapavir (GSK-6207), an indazole derivative, is a long-acting first-in-class approved CA inhibitor. Based on its high lipid solubility and permeability, lenacapavir can be administered orally with an up-to-weekly dosing interval or bi-annual subcutaneous injectable solution. As a CA inhibitor, lenacapavir selectively binds to the hexamer subunits of the HIV-1 CA protein, which is generated by the cleavage of the Gag by viral protease. Upon targeting the CA protein, lenacapavir enables the inhibition of HIV-1 assembly, appropriate viral CA generation, and nuclear import of viral DNA [134,135,136,137,138,139].

Lenacapavir showed a potent in vitro anti-HIV-1 activity against HIV-1-infected MT-4 cells with a mean half-maximum effective concentration (EC_50_) of 105 pmol/L. Lenacapavir manifested a well-tolerated and safe profile with injection site reactions the most frequent off-target event in clinical trials. In a phase 1b study, substantial antiviral activity was observed with lenacapavir. In the phase 3 CAPELLA trial (NCT04150068), lenacapavir was found to reduce HIV-1 viral load in patients with multidrug-resistant infection. A phase 2 study sponsored by Gilead Sciences, Inc. is also ongoing in virologically suppressed people with HIV-1 for investigating the safety and efficacy of lenacapavir in combination with islatravir (an investigational drug and a first potential drug of a new class entitled nucleoside reverse transcriptase translocation inhibitors (NRTTIs)) (Figure 7) (NCT05052996) [140,141,142,143,144,145].

No cross-resistance is reported for lenacapavir with approved antiretroviral drugs. The concomitant use of lenacapavir with strong inhibitors of CYP3A and P-glycoprotein is contraindicated due to the possible low plasma levels of lenacapavir, consequent antiviral activity loss, and the emergence of viral resistance [141,143,145].

### 2.6. Pharmacokinetic Enhancers

**Figure 8:**
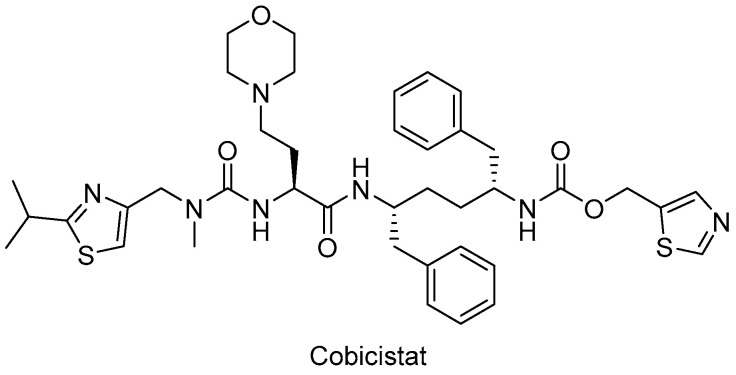
The chemical structure of cobicistat.

In particular, HIV-1 protease inhibitors are prone to metabolize rapidly by CYP3A enzyme predominantly, which can lead to low systemic exposure. Ritonavir, a strong approved HIV protease inhibitor, was found to boost the pharmacokinetics of CYP3A substrate antiviral drugs, including elvitegravir and maraviroc. After the discovery of the CYP3A-inhibitory potency of ritonavir, the strategy of enhancing the pharmacokinetic profile of antiretroviral agents was followed due to the drawbacks of ritonavir use, such as lipodystrophy, hyperlipidemia, insulin resistance, and other undesired drug interactions. Therefore, researchers optimized the chemical structure of ritonavir starting with the removal of the key hydroxyl group and following intensive structure–activity relationship studies and obtained cobicistat (Figure 8), which is more soluble than ritonavir, making coformulation easier. Cobicistat revealed no activity against primary HIV-1 isolates and HIV-1 protease, whereas cobicistat selectively inhibited CYP3A enzyme with minimal cytotoxicity. Cobicistat (Tybost^®^) received its approval from the FDA in 2014 for the treatment of HIV-1 infection. Cobicistat is a pharmacoenhancer of the HIV-1 protease inhibitors atazanavir and darunavir in adults with HIV-1 infection. A fixed-dose combination of cobicistat with darunavir, emtricitabine, and tenofovir alafenamide (Symtuza^®^) is the first protease-inhibitor-based single-tablet regimen approved by the FDA. Cobicistat is also available in two drugs in one pill combinations with darunavir (Prezcobix^®^) and atazanavir (Evotaz^®^). The serum creatinine levels may be augmented with cobicistat use, so caution is required with patients showing kidney problems [90,91,146,147,148,149].

### 2.7. Standard Two-Drug, Three-Drug, and Four-Drug Regimens and Fixed-Dose Combinations as cART Options

Functional antagonism, boosted drug toxicity, and synergistic/additive effects are major possible consequences of combination therapy. Combination therapy has the potential to cause adverse effects due to drug–drug interactions, whereas the decrease in individual drug doses in synergistic drug combinations leads to reduced drug toxicities. Targeting multiple pathways responsible for the disease using different classes of potential drugs contributes to an increase in desired activity [41]. 

The administration of fixed-dose combination therapies and single-tablet regimens for HIV-1 treatment have enabled dramatic improvements such as attenuation of the pill burden, prevention of drug resistance, and improved patient compliance. The combination of lamivudine and zidovudine (Combivir^®^) is the first fixed-dose FDA-approved combination therapy containing two NRTIs (in 1997) for HIV-1 infected people followed by emtricitabine and tenofovir disoproxil fumarate and by abacavir sulfate and lamivudine [150,151]. On the other hand, the combination of dolutegravir and rilpivirine (Juluca^®^) is the first (in 2017) FDA-approved fixed-dose two-drug single-tablet regimen from two different classes of cART (INSTIs and NNRTIs, respectively) for the treatment of adults with HIV-1 infection [152,153]. 

The introduction of protease inhibitors to combination therapy made a huge impact on the management of HIV-1 infection. The current guidelines recommend three-drug combinations with a dual-NRTI backbone and a core drug from the boosted protease inhibitors such as darunavir/ritonavir, integrase inhibitors, or NNRTIs for the initial HIV-1 treatment. Bictegravir, dolutegravir, elvitegravir, and raltegravir (from the INSTIs) and rilpivirine and efavirenz (from the NNRTIs) are usually in the frame for the initial three-drug regimen. Tenofovir disoproxil fumarate plus emtricitabine (in fixed-dose combination) and abacavir plus lamivudine (in fixed-dose combination) are the most preferred NRTIs as a backbone [36,38]. The triple-drug regimen of bictegravir, emtricitabine, and tenofovir alafenamide (Biktarvy^®^) received approval from the FDA in 2018 for the treatment of HIV-1 infected adults. Weight gain, which could result in a high risk of diabetes and cardiovascular disorders, was observed as a disadvantage of this treatment regimen. As HIV-1 and tuberculosis co-infection was frequently encountered, this regimen was found to be safe to be administered along with isoniazid and rifapentine, in contrast to rifampicin, as it induced CYP3A and P-glycoprotein significantly, which could lead to drug–drug interactions [133,154,155].

However, the three-drug regimen is also questioned due to the toxic effects and cross-resistance stemming from the NRTI class. Therefore, dual-therapy regimens stand out as a plausible alternative as a first-line therapy in treatment-naive patients, who cannot tolerate three-drug combination therapy, mainly due to toxicity issues of tenofovir disoproxil fumarate and abacavir. On the other hand, four-drug regimens were considered to obtain benefits from the superior features of INSTIs and HIV-1 protease inhibitors, including swift viral suppression and a higher genetic barrier to resistance, respectively. However, no significant differences in safety or efficacy as well as toxicity or adherence were observed with a four-drug regimen compared to a three-drug regimen [36,38,39,151,156,157,158,159].

Long-acting formulations such as implants, rings, and nanoformulations are also future hallmarks of anti-HIV-1 drug development. The co-packaged cabotegravir (extended-release injectable suspension) and rilpivirine (extended-release injectable suspension) (Cabenuva^®^) is the first FDA-approved injectable complete regimen for adults with HIV-1 infection [160,161].

## 3. The Other Anti-Gag Compounds 

The HIV-1 Gag coordinates all major steps of the assembly of viral particles and it is the only viral protein required to initiate and complete the budding and release of virus-like particles (VLPs) from the plasma membrane [162,163]. The recent discovery and approval of lenacapavir pointed out that targeting Gag leads to the inhibition of HIV-1 assembly, budding, release, and maturation steps and consequent infection of other immune cells.

### 3.1. MA Inhibitors

**Figure 9:**
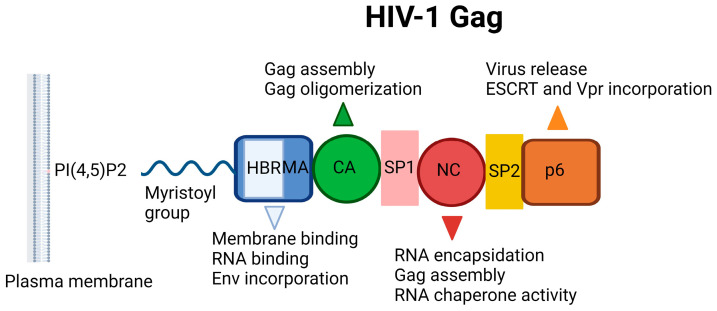
Schematic illustration of HIV-1 Gag. HBR: Highly basic region, MA: Matrix, CA: Capsid, NC: Nucleocapsid, ESCRT: ESCRT: Endosomal sorting complexes required for transport, PI(4,5)P_2_: Phosphatidylinositol-(4,5)-bisphosphate [19]. Image created with BioRender.com.

**Figure 10:**
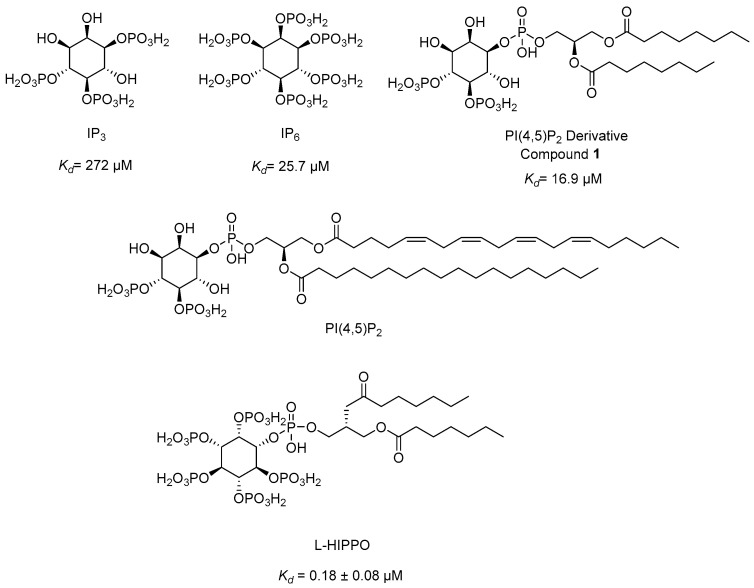
The chemical structures of IP_3_, IP_6_, PI(4,5)P_2_, PI(4,5)P_2_ derivative, and L-HIPPO.

The HIV-1 Gag incorporates MA, CA, NC, and p6 domains, in addition to two spacer peptides, SP1 and SP2, which are located at the CA/NC and the NC/p6 junctions, respectively (Figure 9). The Gag–Gag (multimerization), Gag–membrane, and Gag–RNA interactions are crucial for assembly, mediated by the CA, MA, and NC domains, respectively. The minority of Gag molecules leads to the assembly of a large number of Gag molecules recruiting a single viral RNA dimer to the plasma membrane. The MA domain anchors Gag into the plasma membrane through electrostatic and hydrophobic interactions, but binding is mediated predominantly by dynamic electrostatic interactions. The MA domain is myristoylated on its N terminus and forms ionic interactions with acidic polar heads of phosphatidylinositol-(4,5)-bisphosphate (PI(4,5)P_2_) (Figure 10) through its highly basic region (conserved stretch of lysine and arginine residues). The MA domain is also capable of binding RNA just like NC domain. The specificity of MA binding to PI(4,5)P_2_ might be attributed to MA-RNA binding because this binding prevents Gag from interacting with other lipid interfaces until it reaches the plasma membrane. It was also reported that MA presented a hexamer-of-trimers arrangement for Env incorporation by interacting with the cytoplasmic tail of the Env gp41 protein and some residual mutations hinder this trimeric formation and subsequent Env incorporation for successful virus particle assembly (a stable immature Gag lattice) [162,163,164,165,166,167,168,169,170,171,172,173,174].

The NC domain, comprising two zinc-finger motifs, contributes to Gag multimerization, binds signal Ψ (ΨRNA) specifically, and packages genomic viral RNA. The p6 domain deploys the cellular endosomal sorting complex required for ESCRT machinery, a key component for the release of the budding viral particle from the cell surface [175,176,177,178].

Our research group developed a new surface plasmon resonance (SPR)-based technique in 2010 to detect the binding affinity between Gag/MA binding and various phosphoinositide derivatives containing a different number of phosphates and their regioisomers with or without an acyl chain. We identified that both the divalent phosphate groups and the acyl chains of PI(4,5)P_2_ [164] were essential for strong binding to MA [179]. Then, we carried out SPR analysis of the MA binding of highly phosphorylated inositol phosphates and confirmed that inositol hexakisphosphate (IP_6_) (Figure 10) bound to MA 10-fold strongly than IP_3_ (*K_d_* = 272 µM) (Figure 10) with a dissociation constants (*K_d_*) value of 25.7 µM and comparable to PI(4,5)P_2_ derivative (**1**) (*K_d_* = 16.9 µM) (Figure 10), pointing out the importance of the presence of more phosphate groups in the inositol ring. Strikingly, the dissociation constant of IP_6_ was found to be concordant with the PI(4,5)P_2_ derivative due to the absence of the diacylglycerol moiety in its chemical structure. This outcome encouraged us to design new phosphoinositides carrying a diacylglycerol moiety in the IP_6_ framework. Among the new phosphoinositides, compound **2** (later DL-Heptanoylphosphatidyl Inositol Pentakisphosphate, DL-HIPPO) revealed a remarkable affinity (*K_d_* = 0.25 µM), which was 70-fold and 100-fold stronger than compound **1** and IP_6_, respectively [180]. In a continuation of our work, the potent isomer L-HIPPO (Figure 10) was separated initially and *K_d_* for MA binding of L-HIPPO was found to be 0.18 ± 0.08 μM [181]. Then, we recorded the diffraction patterns of MA-IP_6_ microcrystals beyond 3.5 Å resolution using X-ray free-electron laser (XFEL) study [182]. We elucidated three high-resolution crystal structures of the MA domain in complex with IP_6_ molecules, a part of L-HIPPO (PDB IDs: 7E1I, 7E1J, and 7E1K) [183], by both synchrotron cryo X-ray crystallography and ambient-temperature serial femtosecond X-ray crystallography (SFX) at an XFEL. We determined the important residues in IP_6_ binding and confirmed that the binding site of MA-IP_6_ is distinct from the PI(4,5)P2-binding site and IP_6_ was found to be important in the oligomerization of MA [183]. Afterwards, we carried out computational studies for new rationally designed L-HIPPO derivatives via Maestro software (Schrödinger Release 2016-2) in the MA domain (PDB IDs: 7E1I, 7E1J, and 7E1K) and revealed that benzene-inserted compounds presented a more favorable binding profile than L-HIPPO. Therefore, we applied the same docking procedure to a large library of aromatic groups carrying L-HIPPO derivatives and identified 3,4-dihydroxyphenyl and 3-methoxy-4-hydroxyphenyl carrying compounds as the most potent L-HIPPO derivatives for MA binding with optimum pharmacokinetic properties [184].

**Figure 11:**
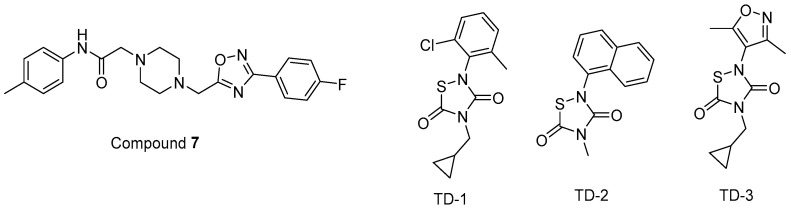
The chemical structures of compounds **7** and TD **1**–**3**.

In other studies, Zentner et al. 2013 [185] developed compound **7** (Figure 11) using a virtual screening method. They further reported that this compound showed potential anti-HIV-1 activity with IC_50_ values of 7.5–15.6 µM. It was also determined that compound **7** directly interacted with HIV-1 MA, competing with PI(4,5)P_2_ for MA binding and blocking the generation of new viruses.

Alfadhli et al. 2013 [186] targeted MA-RNA binding, since RNA binding might protect MA from interacting with other cellular membranes before Gag delivery to the cell surface. They determined four compounds, including three thiadiazolanes (TD **1**–**3**) (Figure 11), that compete with RNA for MA binding. Although thiadiazolanes were found to halt HIV-1 replication, they suffered from toxicity. 

### 3.2. Maturation Inhibitors

The viral protease cleaves Gag and Gag-Pol polyprotein into their mature subunits over the course of the maturation process, leading to dramatic structural rearrangements within the particle. In the final maturation step, the cleavage of six-helix bundle structure by protease sorts out CA from the CA-SP1 cleavage site, disrupts the immature lattice, and frees CA. Then, CA monomers build up the hexameric and pentameric complex of the mature core, which is crucial for maturation and infection. The CA monomers incorporate the N-terminal (CA-NTD) and the C-terminal (CA-CTD) domains, which are separated by a short flexible linker. IP_6_ interacts with the six-helix bundle structure and stabilizes the immature Gag lattice. IP_6_ is also very crucial for the assembly of CA into the mature fullerene-like cone lattice [187,188,189,190,191,192].

Developing maturation inhibitors, which target the cleavage of the SP1 peptide from the CA-SP1 maturation intermediate, has been a bona fide approach to HIV eradication [193]. 

**Figure 12:**
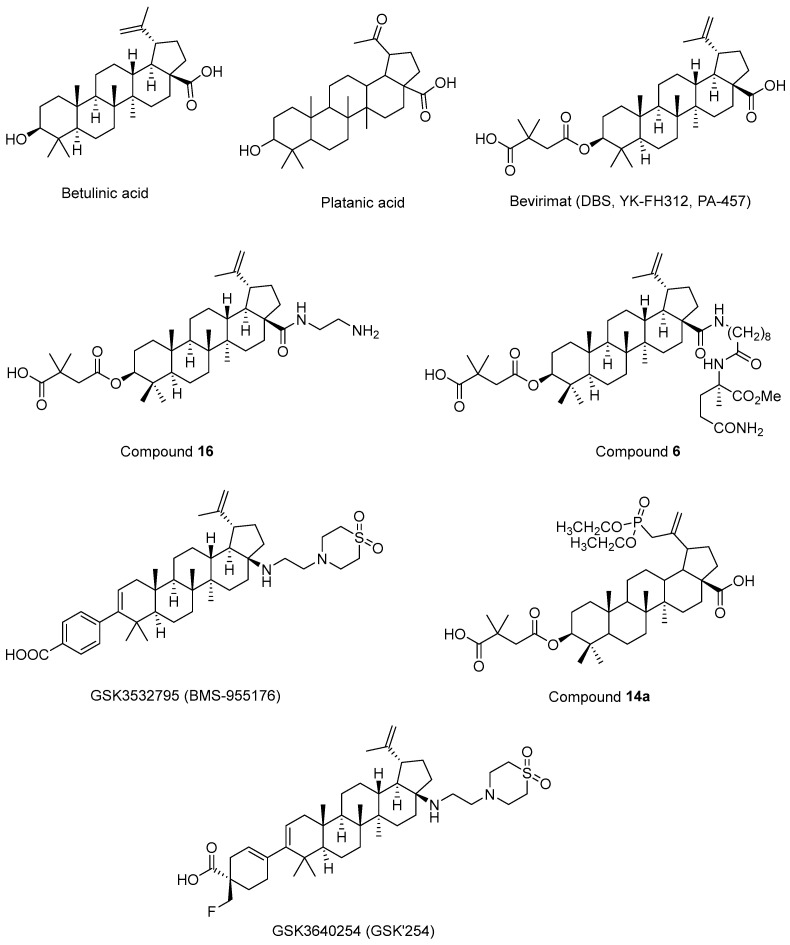
The chemical structures of bevirimat and its potential analogues as anti-HIV-1 agents.

Fujioka et al. 1994 [194] isolated betulinic acid (Figure 12) and platanic acid (Figure 12) from *Syzigium claviflorum* and reported that both of them exhibited HIV-1 replication inhibitory activity against H9 lymphocyte cells. They also reported that dihydrobetulinic acid inhibited HIV replication in same cells with EC_50_ and IC_50_ values of 0.9 µM and 13 µM, respectively. Then, the same research group prepared betulinic acid and dihydrobetulinic acid derivatives and reported that 3-O-(3′,3′-dimethylsuccinyl)betulinic acid (later called DBS, YK-FH312, PA-457, Bevirimat) (Figure 12) exhibited the most potent anti-HIV activity in acutely infected H9 lymphocytes [195]. Kanamoto et al. 2001 [196] demonstrated the virus-induced cytopathic effects of bevirimat in HIV-1IIIB-infected MT-4 cells with an EC_50_ value of 0.011 μg/mL and a CC_50_ value of 14.03 μg/mL. Further mechanistic effects suggested that the formation of viral proteins continued, though the virion could not be released, pointing out that bevirimat affected viral maturation. On the other hand, Li et al. 2003 [197] showed that bevirimat inhibited the replication of wild-type and drug-resistant HIV-1 isolates along with the inhibition of conversion of CA precursor to mature CA. Subsequent research studies also confirmed that bevirimat prevented the cleavage of SP1 from the C-terminus of CA, leading to defective core condensation and inhibition of maturation [198,199,200,201,202]. Although phase I and II studies of bevirimat indicated that bevirimat posed a well-tolerated profile and alleviated the viral load in a dose-dependent manner without drug resistance mutations [203], a later study evaluating the baseline susceptibility to bevirimat found that diminished bevirimat sensitivity was correlated with naturally occurring polymorphisms at 6–8 positions in Gag SP1 [204]. Another bevirimat derivative was prepared and evaluated for anti-HIV effects. Compound **16** (Figure 12) was found to be more effective with a higher hydrosolubility compared to bevirimat. They also characterized a direct interaction of compound **16** and the CA-SP1-NC domain [205]. Dang et al. 2013 [206] synthesized new bevirimat analogues to cope with the resistance issues of bevirimat and evaluated bevirimat resistant HIV-1 variants. Compound **6** (Figure 12) was defined as the most potential analogue against wild-type virus and the bevirimat-resistant NL4-3/V370A variant with IC_50_ values of 0.01 μM and 0.16 μM, respectively. Another bevirimat analogue, GSK3532795 (BMS-955176) (Figure 12), was developed as a potent CA/SP1 cleavage inhibitor, revealing a wide range of antiviral effects including V370A- and ΔV370-containing polymorphic viruses with a low serum binding [207,208,209]. In a phase IIa trial, GSK3532795 was generally found to be safe and well-tolerated and demonstrated a >1 log10 reduction in viral RNA [210]. Gastrointestinal intolerability and treatment-development resistance were detected with GSK3532795 associated with an NRTI backbone in a randomized phase IIb trial [211]. Chroback et al. 2019 [212] synthesized and investigated phosphate and phosphonate analogues of bevirimat for anti-HIV-1 activity. According to the results, compound **14a** (Figure 12) demonstrated similar and more selective anti-HIV-1 activity compared with bevirimat (IC_50_ = 0.03 ± 0.009 μM; Selectivity Index (SI) = 967) with an IC_50_ value of 0.02 ± 0.01 μM and SI value of 3450). They confirmed by computational studies that the phosphonate group contributed to strong interactions of compound **14a** in CTD of CA-SP1. Dicker et al. 2022 [213] developed GSK3640254 (GSK’254) (Figure 12) through a medicinal chemistry approach. They showed that GSK’254 displayed remarkable anti-HIV-1 effects towards a panel of HIV-1 clinical isolates, with a mean EC_50_ value of 9 nM. In phase I studies, GSK’254 was confirmed to possess a favorable clinical potential alone or in combination with tenofovir alafenamide/emtricitabine or dolutegravir. In a phase IIa clinical study, GSK’254 blocked cleavage of p25 in a range of polymorphic HIV-1 Gag VLPs. Phase IIb trials are currently ongoing for GSK’254 (NCT04493216 and NCT04900038).

**Figure 13:**
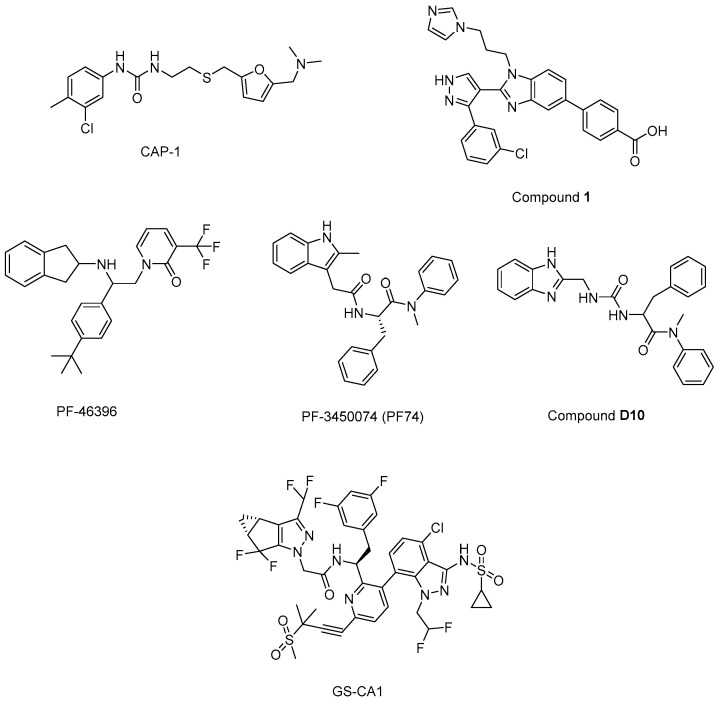
The chemical structures of potential maturation inhibitors.

*N*’-(3-chloro-4-methylphenyl)-*N*-{2-[({5-[(dimethylamino)-methyl]-2-furyl}-methyl)-sulfanyl]ethyl}urea) (CAP-1) (Figure 13) decreased infectivity in latent infected U1 cultures and MAGI cells. Moreover, it was reported that CAP-1 inhibited no early-phase events, while it inhibited late-phase viral events [214]. In the continuation of the research to determine the mechanism of CAP-1 inhibition, a combination of X-ray crystallography and NMR spectroscopy were performed, which indicated the displacement of Phe32 in CAP-1 binding and put emphasis on the significant role of a Phe32 conformational change during normal CA assembly [215].

During the journey to exploration and optimization of new benzodiazepines and benzimidazoles, researchers reached for compounds with potent antiviral activity against wild-type and drug-resistant HIV-1 and both series of inhibitors were able to bind to the N-terminal domain of CA. Then, authors reported the binding of a novel CA-assembly inhibitor targeting an authentic inhibitory site on CA-NTD. The use of compound **1** (Figure 13) as a tool enabled ternary co-crystallizations with CA-NTD [216,217,218,219,220]. 

In another study, a CA assembly inhibitor (CAI), a 12-mer peptide (sequence: IT FEDLLDYYGP-amide), was found to be embedded in a conserved hydrophobic groove and changed the CA dimer interface (CAI binding site), indicating a new target for anti-HIV-1 drug discovery. This peptide was identified as the first known immature HIV-1 assembly inhibitor [221]. The authors later expanded the study and designated *i, i* + 7 stapled peptides and identified NYAD-36 (sequence: Ac-ISF-R8-ELLDYY-S5-ESGS-amide), NYAD-66 (sequence: Ac-ISF-R8-ELLDYY-S5-ED-amide), and NYAD−67 (sequence: Ac-ISF-R8-EWLQAY-S5-EDE-amide) as three potent inhibitors that could bind to CA robustly and collapse the formation of mature-like particles [222]. 

Another new small-molecule inhibitor that targeted virion maturation was introduced from an HIV-1 antiviral screen. PF-46396 (Figure 13), a lead molecule, exhibited potential anti-HIV-1 activity. This compound inhibited the processing of CA-SP1, giving rise to the aggregation of CA/SP1 precursor proteins and maturation inhibition [223]. The same research group then reported PF-3450074 (PF74) (Figure 13) to be effective against all strains of HIV-1 tested with median EC_50_ values of 0.207 µM (range 0.113 to 0.362 µM). A co-crystal structure of PF-74 revealed a new binding site on HIV-1 CA. Moreover, PF-74 in vitro enhanced the rate of HIV-1 CA multimerization [224]. Dostálková et al. 2020 [225] reported a series of modifications of PF74 derivatives. They obtained compound **D10** (Figure 13) with a modified indole moiety to benzimidazole moiety exerting in vitro stabilization activity in higher levels compared to the original PF74 molecule. Researchers continue further modifications to decrease the **D10** cytotoxicity.

Yant et al. 2019 [226] reported GS-CA1 (Figure 13) as a novel small-molecule CA inhibitor that showed remarkable and selective anti-HIV effects with an EC_50_ value of 240 ± 40 pM and CC_50_ > 50 µM against MT-4 cells acutely infected with HIV-1IIIB. Further mechanistic studies indicated that GS-CA1 directly interacted with CA and affected the CA-mediated nuclear import of viral DNA. Moreover, GS-CA1 presented favorable metabolic stability and low solubility to function sustained drug release in mice.

## 4. Strategies for Eradication of HIV-1

The remaining latent reservoirs are host cells containing transcriptionally silent but potentially inducible replication-competent proviruses. The latent reservoirs possess a half-life of approximately 44 months, indicating their durability and making anti-HIV-1 treatment incurable. During the asymptomatic phase of infection, a small portion of latent reservoirs reside in resting CD4^+^ T cells (<10^7^ cells). During cART, the major HIV cellular reservoirs are memory CD4^+^ T cells, whose longevity is very long, and memory CD4^+^ T cells harbor nearly the entire replication-competent HIV reservoir (>95%) in comparison with other CD4^+^ T-cell subsets. The HIV-1 reservoirs are mainly observed in the gastrointestinal mucosa, CNS, lymph nodes, and genital tract, where viral replication becomes autonomous and undetectable [227,228,229,230,231,232,233,234,235,236].

Gene editing technologies including clustered regularly interspaced short palindromic repeat (CRISPR)-associated nuclease 9 (Cas9) [237], immunological approaches such as active immunization with vaccines and passive immunization with broadly neutralizing antibodies for controlling HIV-1 pandemic globally [238], and chimeric antigen receptor cell technology for prolonged remission of the reactivated latent viral reservoirs [239] are recent advancements in HIV treatment. There are also different strategies targeting HIV-1 latently infected cells. These strategies are “shock and kill”, “block and lock”, and “lock-in and apoptosis”. Among them, “shock and kill” is the most advanced one because some agents have entered clinical tests, though the results were not good. Accordingly, a promising new strategy signifies that premature protease activation leads to pyroptotic killing of infected cells for the eradication of the latent reservoir due to toxicity of HIV-1 protease [81]. 

### 4.1. “Shock and Kill (Kick and Kill)” and “Block and Lock”

There are no definitive markers to identify latent reservoirs and there is a need to search for new and better markers that can help in the effort to identify cells with latent HIV-1 proviruses, leading to the elimination of the HIV-1 reservoir. Clonal expansion, known as latently infected cell proliferation, also needs to be addressed, as it is considered one of the main reasons for HIV-1 persistence during cART. Although several approaches such as latency reversal, latency enhancement, immunotherapy with broadly neutralizing antibodies and HIV-1-specific chimeric antigen receptor-T cells, and gene editing technologies that disrupt the provirus have been proposed to eliminate HIV latency, these studies have encountered several high risks and limitations, so there is no broad consensus on the methodology yet [240,241,242,243].

**Figure 14:**
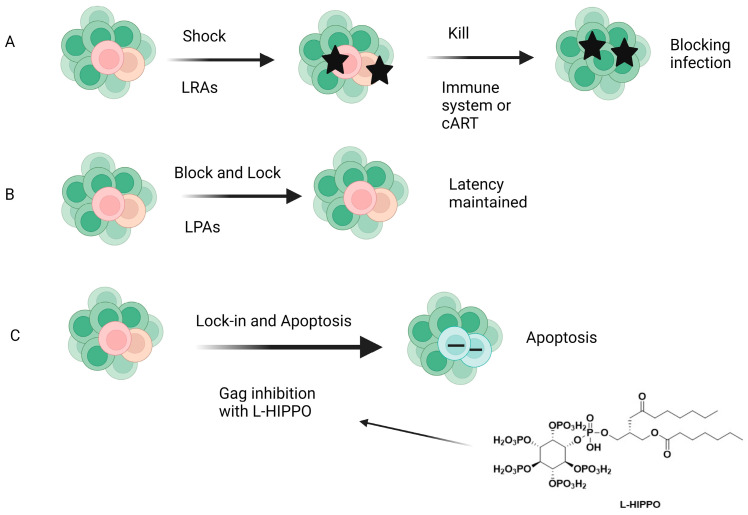
Different strategies for HIV-1 cure. (**A**) Shock and kill, (**B**) block and lock, (**C**) lock-in and apoptosis. LRAs: Latency-reversing agents, LPAS: latency-promoting agents, L-HIPPO: L-Heptanoylphosphatidyl inositol pentakisphosphate [247]. Image created with BioRender.com.

The “shock and kill” (Figure 14) therapy targets the removal of latent proviral reservoirs with the activation of transcription of dormant provirus using pharmaceutical agents (shock) and elimination of the latently infected cells with cART, intrinsic cell death mechanisms, immune responses such as activation of CD8^+^ T cells, and/or HIV-1-cytolysis (kill). Latency-reversing agents (LRAs), mainly functioning as either protein kinase C (PKC) activators, histone deacetylase inhibitors (HDACis), toll-like receptor (TLR) agonists, or PI3K/Akt agonists, have been introduced for this purpose, but none of them have achieved ultimate success. LRAs also have several limitations, such as non-specificity (off-target effects may be present) and the inability to reach all latent reservoirs. On the other hand, the block and lock strategy (Figure 14), which inhibits HIV transcription by targeting both cellular and viral factors with latency-promoting agents (LPAs), aims to achieve durable HIV transcription inhibition. Additionally, the “block and lock” strategy can target both replication-competent and -incompetent viruses, making it noteworthy because replication-deficient viruses can produce viral transcripts and toxic proteins. The “block and lock” strategy could be promising for a functional HIV-1 remission or therapy enabling long-lasting HIV-1 suppression, even independent from cART use. Although a few agents such as Tat or Rev inhibitors were found to suppress the provirus expression, no clinical data have been reported so far [244,245,246,247,248,249].

### 4.2. A New Approach: “Lock-In and Apoptosis” and Compound L-HIPPO

The early clinical trials about the aforementioned strategies failed to reach a scalable solution for curing HIV-1. Our research group came up with a novel strategy called “lock-in and apoptosis” (Figure 14), referring to L-HIPPO (L-heptanoylphosphatidyl inositol pentakisphosphate) (Figure 10 and Figure 14), which was synthesized by our research group as a potential Gag MA-targeting anti-HIV-1 compound. The “killing process” is a problem in “shock and kill”, and this strategy may overcome this problem. Based on this strategy, L-HIPPO was able to circumvent the budding of proviruses and facilitate locking the virus into the HIV-1-infected host cell, leading to the elimination of secretion of new viral particles, a build-up of viral products, and ultimately, to HIV-1-specific host-cell apoptosis [181].

As mentioned above, the apoptosis induction of L-HIPPO with a carrier (L-HIPPO-α-CDE complex) was determined using fluorescence-activated cell sorting (FACS) and microscopic analysis. The results indicated that HeLa cells and Jurkat T cells transfected with pNL4-3/Gag Venus underwent apoptosis in the presence of L-HIPPO-α-CDE complex without showing toxicity [181]. Conceptually, “lock-in and apoptosis” is a new and interesting strategy, but it is in an early stage. For further research, we need to perform animal experiments using a model with the reservoir to confirm its effectiveness for HIV eradication.

### 4.3. Apoptosis and HIV Latency

**Figure 15:**
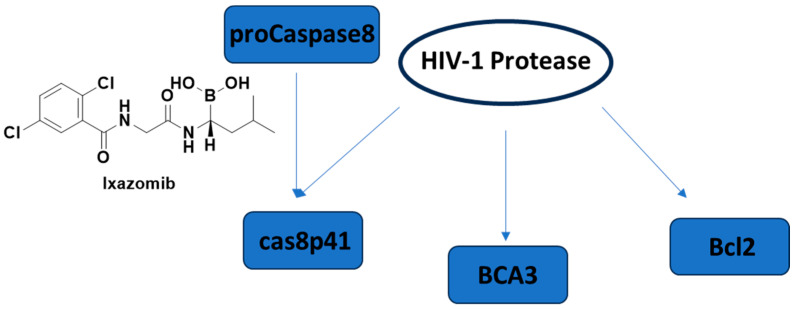
The cell death pathways affected by HIV-1 protease and the FDA-approved proteasome inhibitor ixazomib. BCA3: breast carcinoma-associated protein 3.

Apoptosis, programmed cell death, has already become an emerging concept in HIV-1 latency elimination. HIV-1 protease is also well-known to cleave host proteins in addition to HIV-1 polyprotein at a post-integration step. HIV-1 protease can induce apoptosis by affecting more than one cell death pathway, such as cleaving anti-apoptotic factor Bcl-2, pro-caspase 8, and breast carcinoma-associated protein 3 (BCA3) (Figure 15). The cleavage of caspase 8 leads to the production of a casp8p41 fragment, which can induce apoptosis in a caspase 9- and Bak/Bax-dependent manner, leading to cytochrome c release from mitochondria and activation of caspases 3 and 7. It is noteworthy that the HIV-1 protease cleavage site of pro-caspase-8 is different than the typical cleavage site of protease, highlighting that there is a low possibility of correlated mutation [250,251,252,253,254].

Cummins et al. 2021 [255] conducted a trial of once-weekly oral ixazomib (Figure 15), an FDA-approved proteasome inhibitor, in ART-suppressed, HIV-1 carrying adults. They showed the safety of ixazomib for 24 weeks in HIV-1-infected persons. Latency reversal and decline in HIV-1 reservoir size were observed ex vivo and in vivo. 

## 5. Conclusions and Future Perspectives

The HIV-1/AIDS pandemic continues to grow at an alarming rate worldwide. The state-of-the-art progress in cART has made a dramatic change in the life quality and survival of people living with HIV-1. However, the presence of latent reservoirs, the development of drug resistance, nonadherence, and serious side effects have posed the greatest challenges in HIV-1 treatment. It is imperative that HIV-1-carrying patients should administer lifelong multidrug therapy. All the available anti-HIV-1 drugs only decrease the HIV-1 activity in infected cells without definite elimination. If therapy is interrupted, latent reservoirs can reignite new rounds of infection, destroy the immune system of the patient, and cause eventual death. In particular, deciphering the precise mechanisms of HIV-1 latency will allow us to develop new anti-HIV-1 compounds with durable efficacy, tolerability, and safety. 

A new approach proposed by our research group, “lock-in and apoptosis”, pertains to L-HIPPO, a potential anti-HIV-1 compound that is able to block the budding of offspring viruses after the “kick” process and facilitates the virus to be stuck in an HIV-1-infected host cell, which subsequently undergoes apoptosis along with the virus, leading to definite HIV elimination from the body. Intense research efforts and several modern modalities have been devoted to finding more effective anti-HIV drugs with well-matched pharmacokinetic profile, metabolic stability, improved adherence, and a high barrier to resistance, along with eliminating the latent reservoirs, the insurmountable problem over the years. Up to now, there is no antiretroviral agent that targets the budding phase driven by Gag. At this point, L-HIPPO might be a game-changer filling gaps and accelerating HIV-1 cure.

Abner et al. 2019 [245] once proposed that the additional use of ixazomib could rekindle the hope to be the first true dual-functioning agent, and in our continuing research, ixazomib might contribute to the apoptotic effects of L-HIPPO and rapid elimination of latent reservoirs.

This review aims to interrogate the positive and negative features of the available approved drugs and alternative curative treatment strategies oriented with a purpose of developing novel potent anti-HIV-1 drug candidates with superior properties.
